# Taking the pulse of Mars via dating of a plume-fed volcano

**DOI:** 10.1038/s41467-017-00513-8

**Published:** 2017-10-03

**Authors:** Benjamin E. Cohen, Darren F. Mark, William S. Cassata, Martin R. Lee, Tim Tomkinson, Caroline L. Smith

**Affiliations:** 1 Isotope Geoscience Unit, Scottish Universities Environmental Research Centre (SUERC), Rankine Avenue, East Kilbride, G75 0QF UK; 2 School of Geographical and Earth Sciences, University of Glasgow, Glasgow, G12 8QQ UK; 3 Department of Earth & Environmental Science, University of St Andrews, St Andrews, KY16 9AJ UK; 4 Nuclear & Chemical Sciences Division, Lawrence Livermore National Laboratory, 7000 East Avenue (L-231), Livermore, CA 94550 USA; 5 Department of Earth Sciences, The Natural History Museum, London, SW7 5BD UK

## Abstract

Mars hosts the solar system’s largest volcanoes. Although their size and impact crater density indicate continued activity over billions of years, their formation rates are poorly understood. Here we quantify the growth rate of a Martian volcano by ^40^Ar/^39^Ar and cosmogenic exposure dating of six nakhlites, meteorites that were ejected from Mars by a single impact event at 10.7 ± 0.8 Ma (2*σ*). We find that the nakhlites sample a layered volcanic sequence with at least four discrete eruptive events spanning 93 ± 12 Ma (1416 ± 7 Ma to 1322 ± 10 Ma (2*σ*)). A non-radiogenic trapped ^40^Ar/^36^Ar value of 1511 ± 74 (2*σ*) provides a precise and robust constraint for the mid-Amazonian Martian atmosphere. Our data show that the nakhlite-source volcano grew at a rate of ca. 0.4–0.7 m Ma^−1^—three orders of magnitude slower than comparable volcanoes on Earth, and necessitating that Mars was far more volcanically active earlier in its history.

## Abstract (web summary)

Mars hosts the solar system’s largest volcanoes, but their formation rates remain poorly constrained. Here, the authors have measured the crystallization and ejection ages of meteorites from a Martian volcano and find that its growth rate was much slower than analogous volcanoes on Earth.

## Introduction

**Fig. 1:**
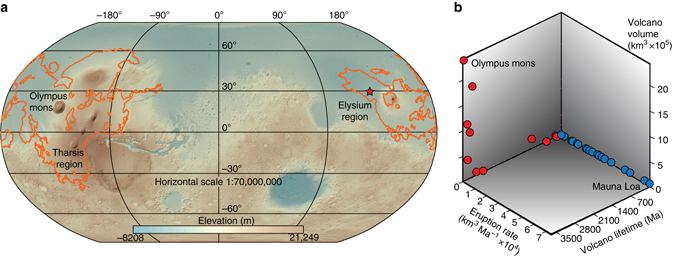
Distribution and size of Martian volcanoes. **a** Amazonian volcanic products on Mars (digital elevation model and *orange outlines* ^43^) are located in the Tharsis and Elysium regions, with the largest volcano, the 600 km wide Olympus Mons, rising more than 21 km above the surrounding plains. The red star in the Elysium region marks the location of a potential source crater for the nakhlites^42^. **b** Relative characteristics of terrestrial and Martian plume volcanism. In comparison to terrestrial volcanoes (*blue*) those on Mars (*red*) have a greater volume and duration, but much lower eruption rates. Data and references in Supplementary Table 1

On Earth, the majority of volcanism and planetary heat loss occurs by plate tectonics, a process driven by mantle convection^1^, with most volcanoes being active along divergent and convergent plate boundaries. A small proportion of terrestrial volcanoes are fed by mantle plumes—highly localized upwellings of abnormally hot rock that ascend from deep within the mantle and undergo partial melting at relatively shallow depths^2^. Mars, on the other hand, has lacked plate tectonics for most of its history^3, 4^. The static ‘stagnant lid’ of the thick Martian lithosphere has restricted convective heat loss^1^, leaving mantle plumes as the dominant, but less efficient, mechanism for transferring heat from the planet’s core to its surface^3, 5^. Tectonic activity continually moves the Earth’s lithospheric plates over mantle plumes, forming age-progressive chains of volcanoes^2^. Even in cases of the slowest-moving plates, individual plume-derived volcanoes on Earth are only active for a few million years^6^. Conversely, Martian plume-fed edifices have remained connected to their magma sources for extraordinary lengths of time—in some cases for billions of years^5, 7, 8^. As a consequence, although morphologically similar to shield volcanoes on Earth, Martian volcanoes have grown to be the largest in the solar system^9^ (Fig. 1).

Radioisotopic dating has been used to determine the lifespans (typically <2 Ma) and eruption rates (typically >1000 m Ma^−1^ and 10,000 km^3^ Ma^−1^) of individual plume volcanoes on Earth^10^ (Fig. 1b), but such approaches have not been applied to Mars. Crater-counting model ages^11^, an approach to estimating the age of a planetary surface based upon the size-frequency distribution of impact craters, indicate that Martian volcanoes have longer lifespans (up to 3500 Ma) but far lower eruption rates (<10 m Ma^−1^ and <700 km^3^ Ma^−1^) (Fig. 1b and Supplementary Table 1). These crater-counting models are useful to resolve the relative ages of planetary surfaces, but are prone to uncertainty in terms of absolute age determination. This is because the crater counting technique makes several assumptions, including the assessment of the differing impactor flux between Mars and the Moon, the extent of crater burial (e.g., by Martian regolith) and the classification of primary vs secondary craters^11, 12^. By instead targeting a unique set of meteorites, the nakhlites, we have sought to accurately determine the age and growth rate of a volcanic system on Mars using high-precision ^40^Ar/^39^Ar dating.

The nakhlites comprise 18 meteorites (not accounting for pairing). Their bulk chemistry is basaltic, with abundant clinopyroxene, less common olivine and a rapidly quenched fine-grained mesostasis comprising feldspar and accessory phases, including chlorapatite and volcanic glass^13^. The nakhlites are also notable for the presence of phyllosilicates and carbonates—products of low-temperature water-rock interaction on Mars^13^. These rocks are mineralogically, chemically and isotopically similar—but not identical^13–15^. Their cosmogenic exposure ages cluster around 11 Ma, indicating the different meteorites spent a similar duration in space while transiting from Mars to the Earth^15, 16^. These data have been employed to suggest the nakhlites were excavated from a localized region of a Martian volcano by a single impact event^13, 16^. During impact and spallation, the nakhlites experienced mild shock metamorphism of 5–20 GPa and ~5–40 °C above ambient^17^. Despite these low degrees of shock heating^17^ and their near-pristine mineralogy^13^, replicate geochronological analyses of the same meteorite disagree; many ages are imprecise and/or fail the robust criteria that define statistical significance (Supplementary Table 2). As such, the temporal relationships between the different nakhlites remain unclear.

In this study, high-resolution (*n* = 43 to 45 steps) laser step-heating ^40^Ar/^39^Ar dating of multiple aliquots (*n* = 2 to 5) of six nakhlites is applied in conjunction with cosmogenic (^38^Ar) exposure dating. The cosmogenic exposure dating allows for accurate correction of cosmogenic and chlorine-derived contributions to the bulk isotope measurements and for determination of accurate ^40^Ar/^39^Ar age data. The six meteorites analysed span the full range of mineralogical variation observed in the nakhlite group^18^. The data show that the nakhlites were not all formed during a single cooling event, but instead reveal a protracted volcanic eruption history on Mars.

## Results

### Argon reservoirs and cosmogenic exposure age

The isotope budgets of meteorites are derived from multiple sources. Martian meteorites contain trapped ^40^Ar, ^38^Ar and ^36^Ar derived from the Martian atmosphere and/or mantle. All meteorites contain variable amounts of cosmogenic ^38^Ar and ^36^Ar, which are produced when high-energy cosmic rays interact with atomic nuclei, primarily during the meteorite's journey from Mars to Earth^16^. Meteorites also contain radiogenic ^40^Ar (^40^Ar*), which is ingrown from the radioactive decay of ^40^K to ^40^Ar, and accumulates once the rocks have cooled below the closure temperature for ^40^Ar diffusion. To calculate accurate ^40^Ar/^39^Ar ages (a measure of the decay of ^40^K to ^40^Ar) it is necessary to establish the contribution of each argon component to the bulk isotope budget of each meteorite^19^.

**Fig. 2:**
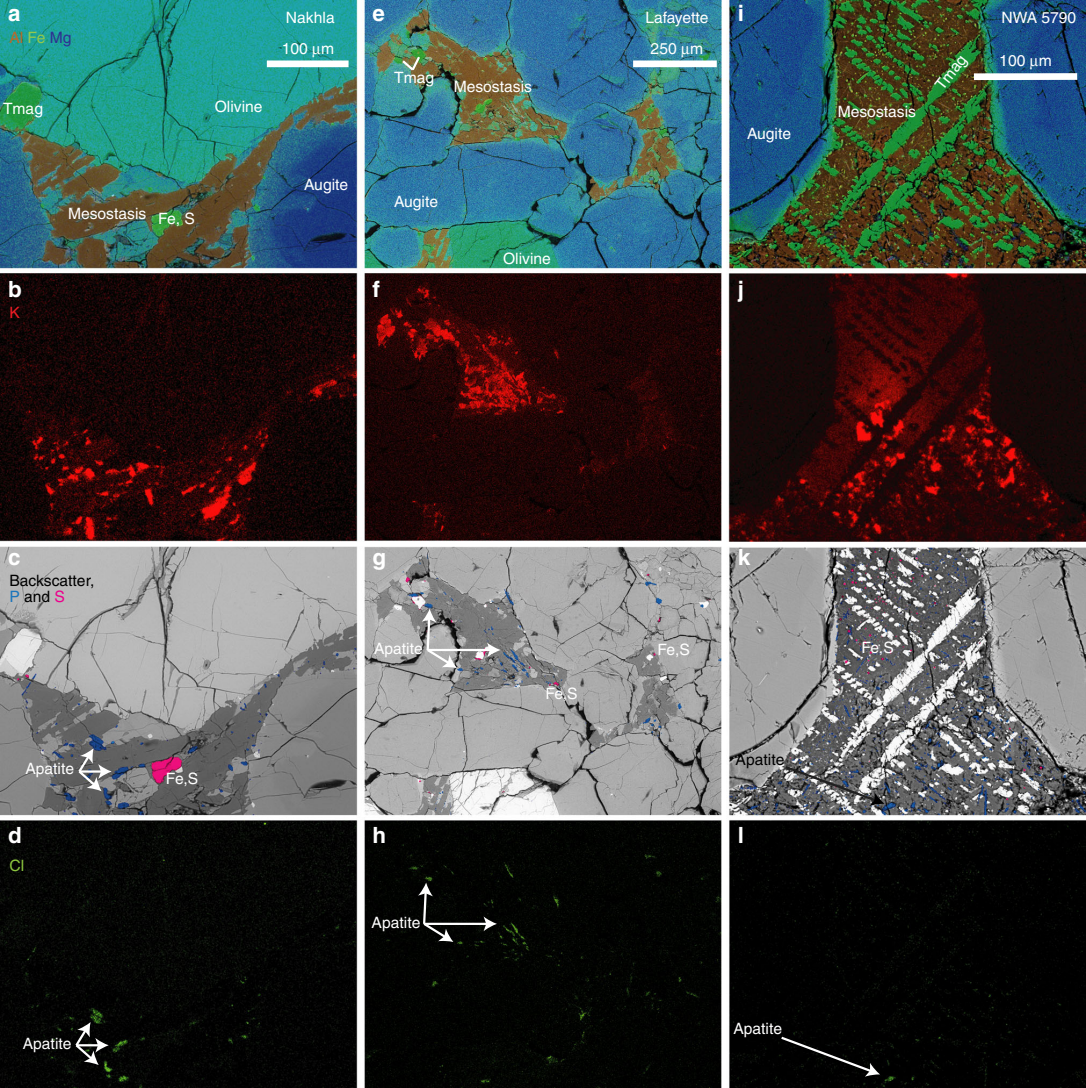
Petrology of nakhlite meteorites. Panels **a**–**d** are scanning-electron microscope images of Nakhla; **e**–**h** are from Lafayette, and **i**–**l** show a representative area of NWA 5790. Images from the other meteorites (MIL 03346, Yamato 000593 and Yamato 000749) are in Supplementary Fig. 1. Panels **a**, **e**, **i** display the distribution of aluminium (*brown*), iron (*green*) and magnesium (*blue*); panels **b**, **f**, **j** show potassium (*red*); panels **c**, **g**, **k** illustrate the distribution of phosphorus (*blue*, concentrated in apatite) and sulphur (*magenta*, in iron sulphide), as well as the backscattered electron intensity in *grey*; and panels **d**, **h**, **l** show the distribution of chlorine (*green*, in apatite). These images illustrate that the nakhlite meteorites are igneous rocks, dominated by euhedral augite crystals set in a fine-grained mesostasis. The augite crystals (*deep blue*, in **a**, **e**, **i**) are often zoned, with an outer rim of ferroan pigeonite. Olivine crystals are also present, but less common. Potassium is concentrated in mesostasis feldspar. The mesostasis also contains crystals of titanomagnetite (Tmag), iron sulphide (Fe,S) and chlorine-bearing apatite. As potassium and chlorine are both dominantly hosted in the mesostasis, the argon gas derived from radiogenic decay of potassium and chlorine gas from apatite will be closely associated in location and thermal behaviour during a ^40^Ar/^39^Ar experiment, and the contribution from chlorine must therefore be accounted for during the cosmogenic argon correction procedure^21^

Contributions from cosmogenic argon have traditionally been determined using simplifying assumptions regarding the distribution of cosmogenic nuclides and the abundances of ^38^Ar and ^36^Ar produced from chlorine during neutron irradiation, which can lead to inaccurate correction of age data^19–21^. The chlorine-derived isotopes are a particular issue for the nakhlite group of Martian meteorites, which can contain over 1000 ppm Cl hosted in salts and/or chlorine-bearing apatite that can be found in the meteorite's mesostasis (Fig. 2 and Supplementary Fig. 1)^22^. Our preferred method determines the cosmogenic argon correction using a combination of the meteorite’s cosmogenic exposure age determined independently from an un-irradiated fragment, and the step-wise ^38^Ar and ^36^Ar cosmogenic production rates calculated from the Ca/K values measured on each ^40^Ar/^39^Ar measurement^21^.

**Fig. 3:**
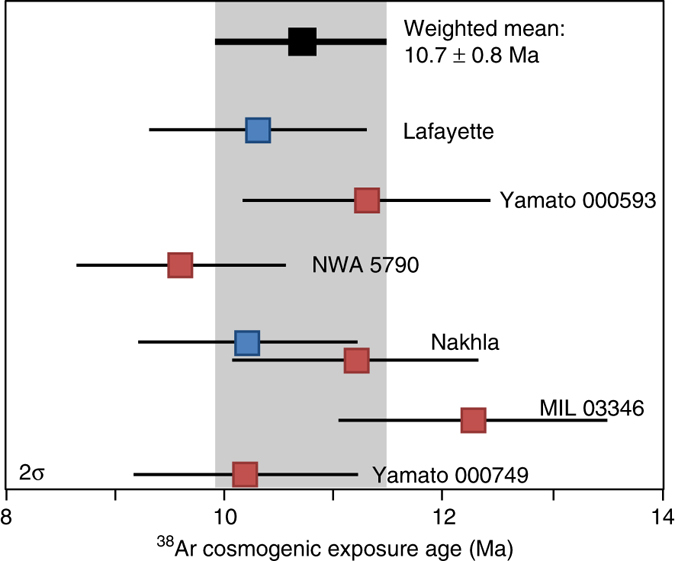
Cosmogenic exposure dates for the nakhlites. ^38^Ar cosmogenic exposure ages overlap within uncertainty, which is consistent with all of the nakhlites being sourced from the same impact. *Blue data points* were analysed at Lawrence Livermore, and *red data points* were analysed at SUERC. The weighted mean cosmogenic exposure age is 10.7 ± 0.8 Ma (2*σ*, full external uncertainty, *bold text* and *grey shaded area*), which represents the timing of the nakhlite impact event on Mars

We analysed unirradiated fragments of each of the six nakhlites, with our data yielding concordant cosmogenic exposure ages, and a weighted mean of 10.7 ± 0.8 Ma (2*σ*, 10% uncertainty on production rates; Fig. 3 and Supplementary Table 3). The indistinguishable cosmogenic exposure ages attest to the fact that the nakhlites can be launch-paired, indicating they were ejected from a single point source on the Martian surface by a single impact event. These cosmogenic exposure ages (Fig. 3 and Supplementary Table 3) were used to correct the ^40^Ar/^39^Ar data as discussed above^21^.

### Martian atmospheric ^40^Ar/^36^Ar composition

**Fig. 4:**
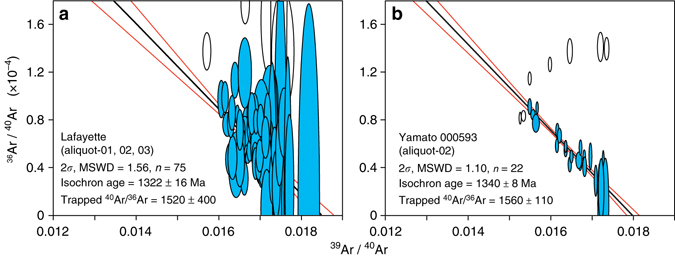
Trapped Martian ^40^Ar/^36^Ar at the time of nakhlite formation. Isochron analysis of **a** Lafayette and **b** Yamato 000593 reveal the composition of initial trapped ^40^Ar/^36^Ar component. All symbols and results are reported at the two-sigma level. *Blue ellipses* denote plateau steps that were included in the isochron regression, *white ellipses* are outliers from the low-temperature steps that were excluded from the isochron analysis, and the *red lines* indicate the 2*σ* uncertainty envelope

An appropriate correction factor for the Amazonian Martian atmosphere was determined via isochron analysis of the ^40^Ar/^39^Ar results. Negligible Marian trapped argon components for most of the nakhlites resulted in little variation in ^36^Ar/^40^Ar (Supplementary Data 1). However, higher yields of trapped argon in aliquots of Lafayette and Yamato 000593 facilitated isochron regression and calculation of accurate values for the ^40^Ar/^36^Ar trapped component. Lafayette yields a trapped ^40^Ar/^36^Ar ratio of 1520 ± 400 (2*σ*) and Yamato 000593 defines a trapped ^40^Ar/^36^Ar ratio of 1560 ± 110 (2*σ*; Fig. 4). These two ratios are indistinguishable, and the latter was used to correct each sample for Martian atmospheric contribution.

### Nakhlite ^40^Ar/^39^Ar geochronology

**Fig. 5:**
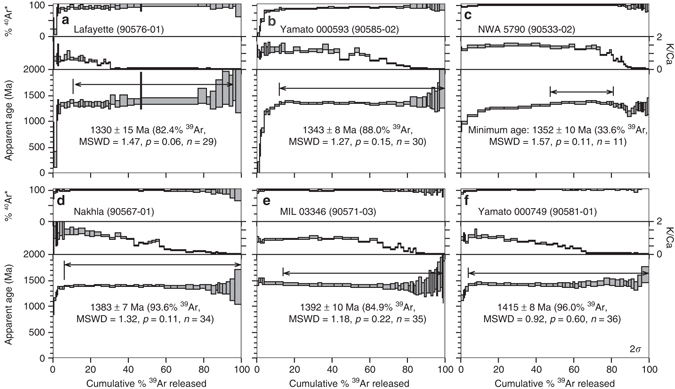
Representative ^40^Ar/^39^Ar age spectra for the nakhlites. Samples are arranged in chronologic order from youngest to oldest: **a** Lafayette aliquot 01, **b** Yamato 000593 aliquot 02, **c** NWA 5790 aliquot 02, **d** Nakhla aliquot 01, **e** Miller Range 03346 aliquot 03 and **f** Yamato 000749 aliquot 01. The *top panel* of each sample represents the portion of radiogenic Ar (^40^Ar*) released from each degassing step; the *middle panel* represents the K/Ca value for each step; and the *lower (main) panel* shows the calculated ^40^Ar/^39^Ar age for each degassing step. All uncertainties are 2*σ*. The nakhlites yield excellent results, often with statistically robust plateaus comprising >90% of the ^39^Ar released. NWA 5790 **c** is an exception, with concordant steps spanning only 34% of the ^39^Ar released; these results therefore provide only a minimum age constraint for the eruption of this sample. Additional aliquots from these meteorites (*n* = 2 to 5) are highly reproducible, with similar degassing spectra and concordant plateau ages (Supplementary Figure 2)

After the above contributions from cosmogenic and atmospheric argon were accounted for, the ^40^Ar/^39^Ar data were plotted on age spectra (Fig. 5 and Supplementary Fig. 2). Plateau ages were defined as contiguous steps that overlap within two-sigma uncertainty, comprising >70% of the ^39^Ar released^23, 24^. Twelve of 19 aliquots met this criteria—with many plateaus including >90% ^39^Ar (Fig. 5 and Supplementary Fig. 2), indicating that the Ar-systematics have been relatively undisturbed. Three aliquots (Lafayette aliquot 02, MIL 03346 aliquot 2 and Yamato 000593 aliquot 3) yielded plateau segments containing between 52 and 59% ^39^Ar, less than the 70% ^39^Ar cutoff for plateau ages (Supplementary Fig. 2). Nevertheless, as these three aliquots have plateau segments that are concordant with other aliquots from the same samples that yielded plateaus with >80% of the ^39^Ar released, we are confident that these three aliquots also record eruption ages. NWA 5790 spectra are more disturbed than the other nakhlites (Fig. 5 and Supplementary Fig. 2). Two of the three aliquots, however, yielded 11 and 13 contiguous steps (ca. 34% ^39^Ar) that overlap within two-sigma analytical uncertainty. As 24 separate analyses from two different aliquots overlap within analytical uncertainty, we conservatively interpret these data to represent a minimum eruption age for NWA 5790.

The eruption age for each meteorite was calculated as a weighted-mean of all plateau steps. Analytical uncertainties were calculated as the standard error of the mean, but if the mean square weighted deviates (MSWD or reduced chi-squared statistic) was greater than one, the analytical uncertainty was inflated by multiplying the standard error of the mean by the square-root of the MSWD. All samples analysed in this study (including NWA 5790) have plateau and weighted-mean ages with MSWD of less than two, and probability of fit (“*p*”) values of greater than 0.05 (95% confidence), indicating the analyses are statistically robust^23, 24^.

Our robust ^40^Ar/^39^Ar data are a product of the high-resolution approach to the step-heating experiments^25^, and employment of procedures^21^ to make appropriate corrections for cosmogenic and chlorine-derived ^36,38^Ar—which is important given the aforementioned abundance of chlorine in the nakhlites (Fig. 2, sometimes >1000 ppm Cl^22^). The highly reproducible age data are consistent with the near-pristine character of the meteorites and associated low degrees of shock metamorphism^17^. The experimental design^25^ also facilitated resolution of the different argon reservoirs in each meteorite (Fig. 2a–c).

## Discussion

The trapped components previously measured in the nakhlites MIL 03346 (^40^Ar/^36^Ar = 1425 ± 230)^26^ and Yamato 000593 (^40^Ar/^36^Ar = 1502 ± 159)^27^, as well as Chassigny (^40^Ar/^36^Ar = 1452 ± 168)^27^ are commensurate with our analyses (Fig. 4). All of these meteorites crystallized at ca. 1300–1400 Ma (Fig. 5 and ref. ^28^), and therefore the common trapped component likely reflects the isotopic composition of the Martian atmosphere at that time. The weighted average of these measurements is 1511 ± 74 (2*σ*) for the ^40^Ar/^36^Ar trapped component—the most precise constraint yet obtained for the mid-Amazonian atmosphere of Mars. This value is more precise than, but within analytical uncertainty of, measurements by the Curiosity rover for the present-day Martian atmosphere (1900 ± 600, 2*σ*)^29^. It is, however, distinct from the paleo-atmospheric ^40^Ar/^36^Ar recorded by the shergottite meteorites (^40^Ar/^36^Ar of 1800 ± 100)^30^, which yield ^40^Ar/^39^Ar cooling ages of ca. 180–600 Ma^21^. Together, these data therefore suggest that the atmospheric ^40^Ar/^36^Ar of Mars has increased significantly (Δ ^40^Ar/^36^Ar of 300 ± 130) over the last 1300 Ma. The ^40^Ar/^36^Ar value of Earth’s atmosphere has likewise increased over time^31^, and reflects degassing of radiogenic ^40^Ar from the interior. In the case of Mars, the change in ^40^Ar/^36^Ar also provides constraints on atmospheric loss through time^32^.

**Fig. 6:**
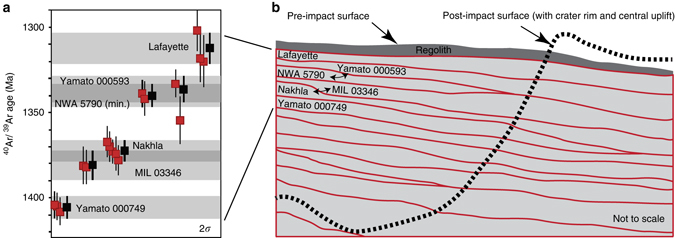
Stratigraphic model for the nakhlite meteorites. **a** Summary of ^40^Ar/^39^Ar age data. Each meteorite has multiple aliquots with highly reproducible plateau ages (*red squares*). *Bold black squares* and *horizontal grey bars* represent weighted mean ages. The ^40^Ar/^39^Ar results indicate that the nakhlites were erupted in at least four temporally discrete eruptions, with volcanic activity spanning 93 ± 12 Ma. All uncertainties are 2*σ*. **b** Schematic cross-section for a layered lava flow sequence, with nakhlite stratigraphic relationships and outline of post-impact structure

The ^40^Ar/^39^Ar ages show the nakhlites were erupted between 1416 ± 7 and 1322 ± 10 Ma (2*σ*, i.e., mid-Amazonian), a period spanning 93 ± 12 Ma (Table 1 and Fig. 6). Our interpretation is that the nakhlites have sampled a layered volcanic sequence, with the ^40^Ar/^39^Ar ages defining stratigraphic position (Fig. 6). Two sets of meteorites (respectively: Nakhla and MIL 03346; NWA 5790 and Yamato 000583) are not temporally resolvable at the 2*σ* level (Fig. 6); however, geochemical and petrographic differences between them^15^ suggest they may also sample separate flows. A layered volcanic sequence is consistent with the subtle but significant mineralogical, petrological, geochemical and isotopic differences between nakhlites^13–15, 33^, which we interpret as due to changes in magma composition between related—but temporally distinct—extrusive units from the same volcano (Fig. 6). This scenario is unsurprising if one considers an analogy of a moderate-sized bolide hitting a plume-fed shield volcano on Earth (e.g., Mauna Kea, Hawai’i) and the number of chemically similar yet temporally distinct igneous units that could be ejected.

**Table 1:** ^40^Ar/^39^Ar ages for the nakhlites

**Meteorite**	**Age (Ma)**	**±2** ***σ*** **(analytical/full external** ^**1**^ **)**	**# analyses in age**	**MSWD**	**Meteorite source & catalogue number**	**Fall/Find information**
Lafayette	1321.7	9.3/9.6	78	1.50	Smithsonian Museum, USA; USNM 1505	Find, 1931, USA, 800 g
Yamato 000593	1346.0	7.6/7.8	48	1.58	Japanese Antarctic Meteorite Research Center; Yamato 000593 (37)	Find, 2000, Antarctica, 13.71 kg
NWA 5790	1350.1	6.8/7.0	24	1.17	Macovich Collection, New York; NWA 5790	Find, 2009, Northwest Africa, 145 g
Nakhla	1382.5	6.2/6.6	118	1.01	Natural History Museum, London; BM 1913,25, 83	Fall, 1911, Egypt, 10 kg
MIL 03346	1390.9	8.6/8.9	51	1.44	The Meteorite Working Group, NASA; MIL 03346, 118 (205)	Find, 2003, Antarctica, 715 g
Yamato 000749	1415.6	6.6/7.0	101	0.88	Japanese Antarctic Meteorite Research Center; Yamato 000749 (59)	Find, 2000, Antarctica, 1283 g

^a^The full external age uncertainty was calculated using a Monte Carlo simulation^48^

Our stratigraphic model is consistent with the geology of Martian volcanoes. High-resolution satellite imagery has revealed sequences of lava flows, with individual layers typically 4–26 m thick^34, 35^, commensurate with the planet’s relatively low gravity that allows for the eruption of numerous thin lava flows that extend for long distances^34^. Our model of a layered volcanic sequence (Fig. 6) differs from a previous interpretation of the nakhlites that invoked sampling from a single thick flow/intrusive unit^18, 36, 37^. Such a model where the nakhlites are from a single thick flow or intrusion requires that all of the meteorites would have the same cooling age, which is inconsistent with our ^40^Ar/^39^Ar data (Fig. 6).

We also note that the two Yamato meteorites have ^40^Ar/^39^Ar ages that differ by 70 ± 10 Ma (2*σ*). This age difference is incompatible with the hypothesis that these were fall-paired stones^38^ (i.e., two parts of a formerly larger meteorite that have become separated, e.g., during atmospheric entry). Despite being found in the same Antarctic field season, there is, however, no a priori scientific reason for why these stones should be fall-paired, particularly when considering that the Yamato meteorite stranding surface is the largest in Antarctica, covering an area of 4000 km^2^, and that the glaciers which feed into the stranding area extend for a further 500 km upstream^39^. Yamato 000593 and Yamato 000749 could therefore have fallen anywhere in the catchment area, only to be brought to a similar part of the ice field by the action of Antarctic glaciers.

Our ^40^Ar/^39^Ar and cosmogenic exposure ages inform about the provenance of the nakhlites on the surface of Mars, including their ejection crater, and properties of their source volcano. More than seven different Martian craters have been suggested as potential sources for the nakhlites^40–42^. However, when we use the recent re-mapping of the Martian surface by NASA^43^, we find that only one of these craters is situated in a mid-Amazonian volcanic terrain compatible with our ^40^Ar/^39^Ar results (Fig. 1 and Supplementary Table 4). This crater, which is located at 130.799°E, 29.674°N, has preserved ejecta rays^42^ that are indicative of a recent impact event, which in turn is consistent with the cosmogenic exposure age (10.7 ± 0.8 Ma) obtained for the nakhlites (Fig. 3). This crater has a diameter of 6.5 km, which is large enough for the impact to have had sufficient energy to have excavated and ejected material beyond Mars’ orbit^44^.

**Fig. 7:**
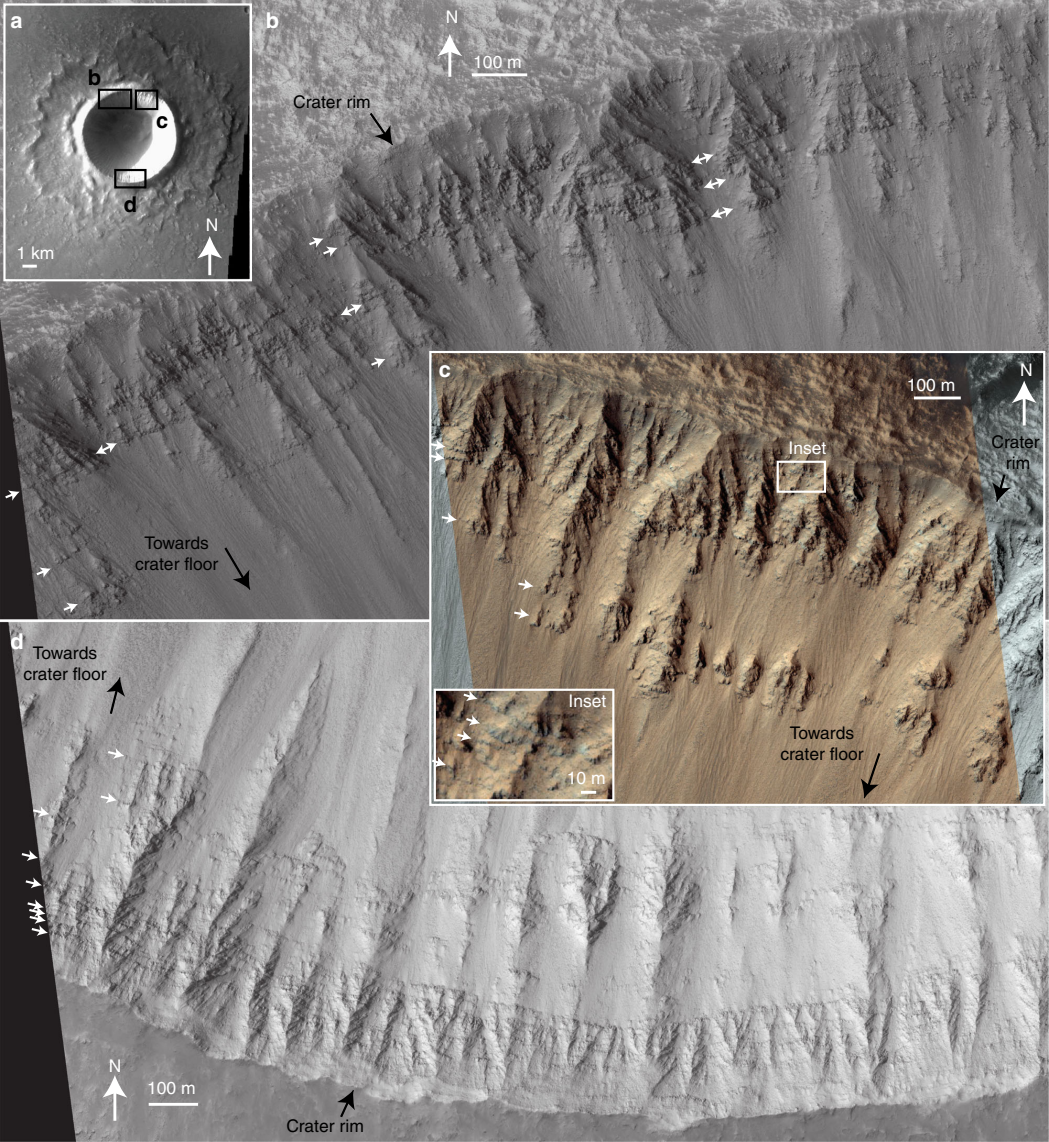
A potential nakhlite source crater. This crater is located on the Elysium lava plains, to the northwest of the Elysium shield volcano on Mars, at 130.799°E, 29.674°N (Fig. 1). **a** Overview of the crater, which is 6.5 km in diameter^42^, large enough to have ejected Martian rocks towards Earth^44^. THEMIS image V13713007, Band 3, NASA/ASU. *Black rectangles* indicate the locations for subsequent images. **b**–**d** Detail of the northwestern, northeastern and southern crater rim, respectively, representing parts of HiRISE image ESP 017997_2100, NASA/JPL/University of Arizona. These images show numerous sub-horizontal layers, which are interpreted as lava flows (e.g., ref. ^35^ for similar features elsewhere on Mars). *White arrows* indicate prominent layers; see inset for detailed view. Solar illumination is from the west in all images

We have investigated high-resolution satellite images from the walls of this crater, which provide clear evidence for multiple layers (Fig. 7); similar layers elsewhere on Mars are interpreted as lava flows^35^. During an impact event the ejecta that exceeds escape velocity comes from the near-surface of Mars, up to a maximum depth of 0.2 times the impactor’s radius^17^. The 6.5 km diameter of this crater^42^ requires the impacting bolide to have a radius of ca. 200 m (Supplementary Table 4). Thus, the spallation zone for this crater is from the near-surface to a depth of 40 m (i.e., 0.2 × 200 m). If the identified crater is not the source of the nakhlites, there are other rayed craters^41^ situated on Amazonian volcanic terrains that have diameters of up to 10 km, which increases the maximum spallation depth to 66 m (Supplementary Table 4).

If the nakhlite samples analysed span the full spallation depth (near-surface to 40–66 m), then considering the time taken to accumulate the lava sequence (93 ± 12 Ma), approximately 0.4–0.7 m of lava was extruded every 1 Ma. If the nakhlites analysed do not span the full spallation depth, this rate represents an upper bound, whereas if the source crater is located on the volcano flank, or on lava plains, then the calculated rate represents a lower bound, as eruptions are likely to be more voluminous and frequent closer to the centre of an edifice. In all scenarios, the growth rate is three orders of magnitude lower than is observed for a terrestrial plume-derived volcano (e.g., 8400 ± 2600 m Ma^−1^ at Mauna Kea, Hawai’i)^10^.

Extrapolating a growth rate of 0.4–0.7 m Ma^−1^ to the full 6–22 km thickness of the massive Tharsis and Elysium volcanoes^7^ would necessitate volcanic activity over a timespan exceeding the age of the solar system (>31,000 Ma). Therefore, the eruption rate must have been greater prior to the mid-Amazonian. Both crater counting and planetary heat-flow models support this conclusion, as they demonstrate that the rate of volcanism during the Noachian and Hesperian (4500–3000 Ma) was at least 2–10 times greater than in the Amazonian (<3000 Ma)^3, 4, 7^. Our data compare favourably with remote-sensing crater-counting studies of Martian volcanoes^45–47^, which indicate that volcanism occurred at much lower rates in the recent past, compared to early in the planet’s history. For example, over the last 300 Ma the maximum effusion rate at the Arisa Mons volcano^47^ was between 1 and 8 km^3^ Ma^−1^—well below the eruption rate of 270 km^3^ Ma^−1^ averaged over the 3400 Ma duration of the volcano (Supplementary Table 1).

Our data thus quantify the growth rate of a mid-Amazonian volcano, and robustly show via radioisotopic dating that the minimum lifespan of at least one Martian edifice (93 ± 12 Ma; Fig. 2) far exceeds that of any terrestrial counterpart. This work also resolves the temporal relationships between the nakhlite meteorites, which sample a series of lava flows (Fig. 6), rather than a single thick intrusion/flow^18, 36, 37^. The high-precision data also provide an ‘absolute’ temporal anchor for the surface of Mars that could underpin a Martian crater counting calibration model, providing the provenance of the nakhlites (i.e., location of their source crater, Fig. 7) can be confirmed.

## Methods

### Qualitative X-ray element mapping

These data (Fig. 2 and Supplementary Fig. 1) were collected via energy-dispersive spectroscopy on a Carl Zeiss Sigma scanning electron microscope at the University of Glasgow. Samples were carbon-coated, and the electron microscope was operated in high-vacuum mode at 20 kV and ~ 2 nA.

### ^40^Ar/^39^Ar chronology

Samples were prepared at the NERC Argon Isotope Facility, Scottish Universities Environmental Research Centre (SUERC). The meteorites were first gently crushed in an agate mortar and pestle to liberate mineral phases, which were then washed in distilled water to remove dust. Hand-picked chips of groundmass were then wrapped in Cu-foil packets, and loaded in two irradiation vials along with neutron-fluence monitor Hb3gr (standard age of 1081.0 ± 1.2 Ma (1*σ*)^48^ and secondary standard GA1550 (standard age of 99.738 ± 0.104 Ma (1*σ*)^48^. Samples were then irradiated for 80 h in the Cd-lined TRIGA facility, Oregon State University, USA. After a decay period of 5–9 months, analyses of neutron flux monitors and meteorite samples were undertaken on an MAP 215-50 spectrometer operated in peak-hopping mode, with measured sensitivity of 1.13 × 10^−13^ mol V^−1^. Hb3gr crystals were analysed via total-fusion, while meteorite samples were incrementally heated using a using a defocused CO_2_ laser. The released gases were purified using two SAES GP50 getters with ST101 Zr-Al cartridges, one at room temperature (0 V) and the other at ~ 450 °C (2 V). Isotope extraction, purification, extraction line operation and mass spectrometry were fully automated. Data were corrected for background measurements, mass discrimination and radioactive decay since irradiation using *MassSpec* software version 8.131. Values used in the ^40^Ar/^39^Ar data regression are listed in Supplementary Table 5.

### Cosmogenic exposure ages

These analyses were performed on unirradiated aliquots, and were measured both at SUERC and Lawrence Livermore National Laboratory (LLNL). Gas extractions at SUERC followed the procedure described above. At LLNL, whole-rock fragments of Nakhla and Lafayette were loaded into small metal packets made from high-purity Pt–Ir tubes and heated with a 75 W diode laser (*λ* = 810 ± 10 nm)^21^. The released argon was purified using four SAES getters (one hot and three cold) and analysed using a Nu Instruments Noblesse mass spectrometer. Samples were analysed statically in peak hopping mode using the axial multiplier detector. Sample analyses were bracketed by analyses of a spike of known abundance and isotopic composition to monitor for time-dependent changes in spectrometer sensitivity and mass discrimination.

Cosmogenic exposure ages were calculated from elemental production rates^49^. When possible, chemical compositions of each sample were determined by inductively coupled plasma mass spectrometry (ICP-MS) at LLNL (Supplementary Table 3). In two instances (Lafayette and Nakhla aliquot ‘b’), average chemical compositions^50^ were used for the specific meteorites (see Supplementary Table 3). For ^38^Ar exposure age calculations, the relative contributions of trapped and cosmogenic Ar were determined by a two-component deconvolution assuming cosmogenic (^38^Ar/^36^Ar = 1.54)^51^ and the trapped component is derived from the Martian atmosphere (^38^Ar/^36^Ar = 0.244)^52^.

To confirm that the small variations in cosmogenic exposure age determined for the different nakhlites (Fig. 3) are not responsible for the differences in ^40^Ar/^39^Ar ages across the group (Fig. 6), we also calculated the ^40^Ar/^39^Ar ages using the weighted mean cosmogenic exposure age of 10.7 ± 0.8 Ma. These calculations (Supplementary Fig. 3) demonstrate that the plateau age for each meteorite is insensitive to small (1–2 Ma) variations in cosmogenic exposure age, and that the ca. 90 Ma age difference between Lafayette and Yamato 000749 is robust (Supplementary Fig. 3).

### Data availability

The authors declare that all data supporting the findings of this study are included in this published article (and its supplementary information files).

## Electronic supplementary material

Supplementary Information

Supplementary Data 1
