# Supplementary material for: Taking the pulse of Mars via dating of a plume-fed volcano
Source: Nat Commun. 2017 Oct 3;8:640. doi: 10.1038/s41467-017-00513-8 (PMC5626741; doi:10.1038/s41467-017-00513-8)
Supplement: Supplementary file 1 — Supplementary Information [file 41467_2017_513_MOESM1_ESM.pdf]

**Description of Supplementary Files**

File Name: Supplementary Information
Description: Supplementary Figures, Supplementary Tables and Supplementary References

File Name: Supplementary Data 1
Description: Full analytical 40Ar/39Ar geochronologic results for each meteorite

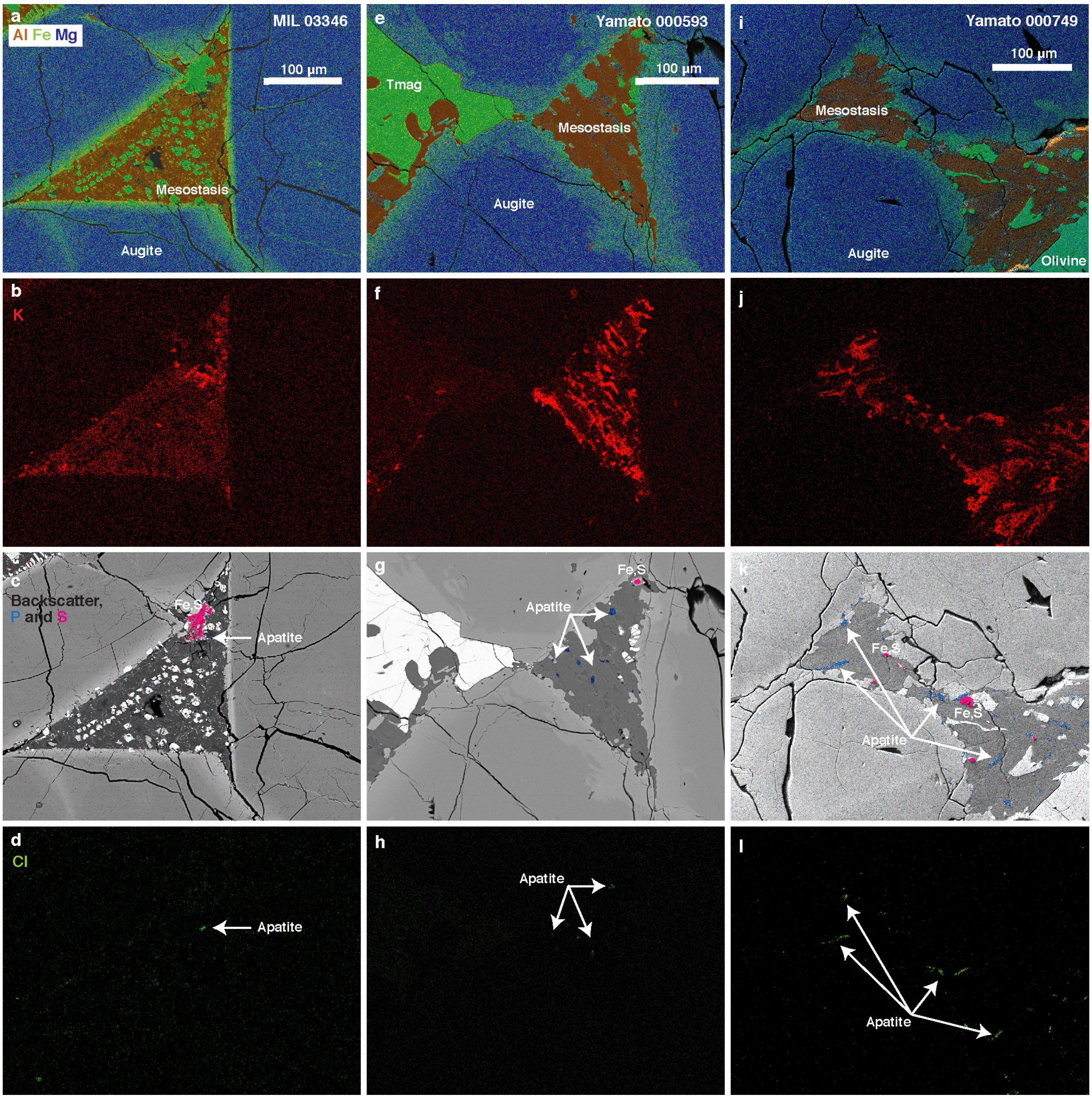


**Supplementary Figure 1**. **Petrology of nakhlite meteorites.** Meteorites in these images are: (**a-d**) Miller Range 03346, (**e-h**) Yamato 000593, and (**i-l**) Yamato 000749. Colours are the same as in Fig. 2 of the main manuscript: (**a**, **e**, and **i)** Al (brown), Fe (green), Mg (blue); (**b**, **f**, and **j**) K (red); (**c**, **g**, and **k**) backscattered electron intensity (grey), P (blue), S (magenta), and (**d**, **h**, and **l**) Cl (green). Euhedral cumulate crystals of augite dominate, and these augite crystals are often zoned, with an outer rim of ferroan pigeonite. Cumulate olivine is also present, but less common. Potassium is concentrated in mesostasis feldspar. The mesostasis also contains crystals of titanomagnetite (Tmag), iron sulphide (Fe, S), and chlorine-bearing apatite. As K and Cl are both dominantly hosted in the mesostasis, gas from the mesostasis phases will be closely associated during a $^{40}Ar/^{39}Ar$ experiment, and the contribution from Cl must therefore be accounted for during the cosmogenic Ar correction procedure[1].

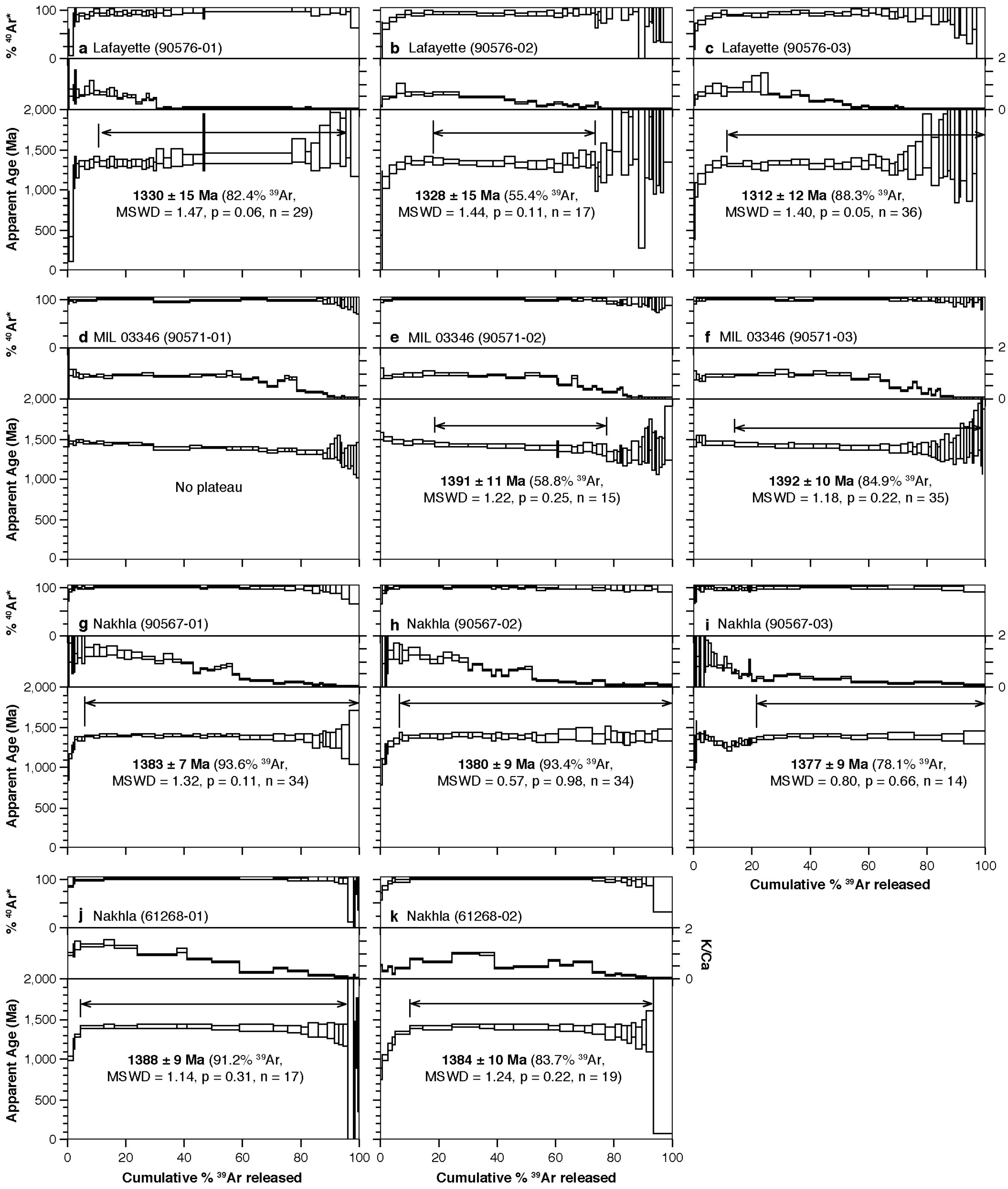


**Supplementary Figure 2**. **$^{40}Ar/^{39}Ar$ results for all samples analysed.** Note that the high-temperature steps often have larger age uncertainties, due to the increased levels of Ca-derived cosmogenic correction involved for these steps[1].

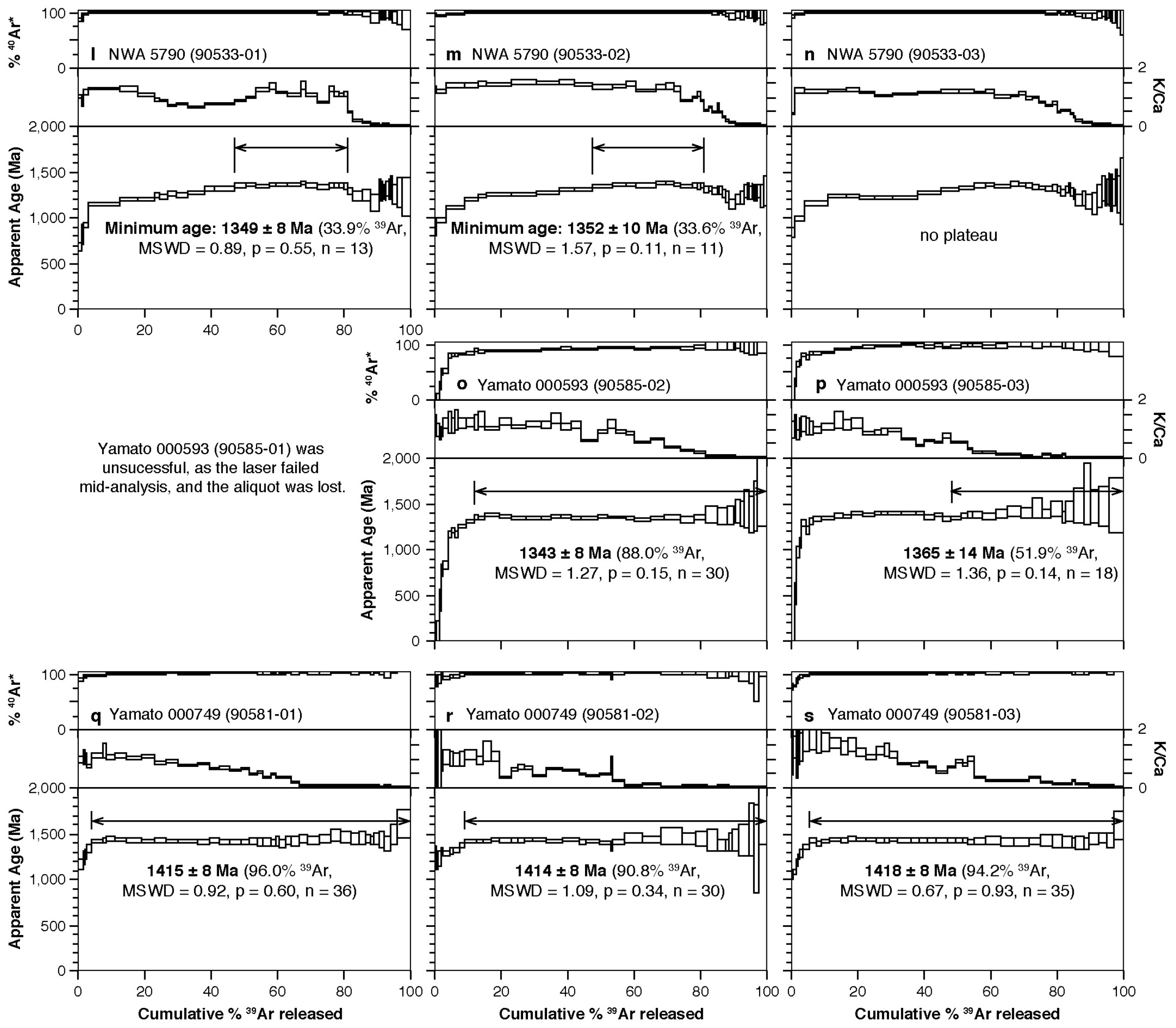


**Supplementary Figure 2 (continued).**

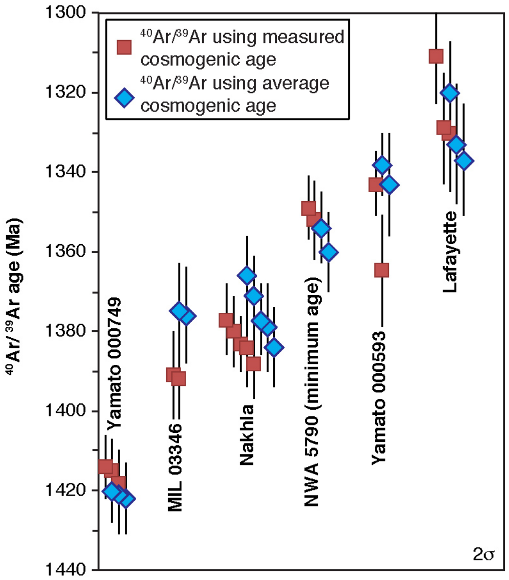


**Supplementary Figure 3**. **Summary of $^{40}Ar/^{39}Ar$ plateau ages for the nakhlites**. To show that the small variations in cosmogenic exposure age determined for the different nakhlites (Supplementary Table 3) are not responsible for the different $^{40}Ar/^{39}Ar$ ages across the group, we have calculated the $^{40}Ar/^{39}Ar$ ages for each sample in two ways. Firstly, using the measured cosmogenic exposure age for each sample (red squares: i.e., the ages reported in the manuscript; Table 1), and secondly using the weighted mean cosmogenic exposure age of 10.7 ± 0.8 Ma (blue diamonds). These calculations demonstrate that the plateau age for each meteorite is fairly insensitive to small (1–2 Ma) variations in cosmogenic exposure age, and that the ~90 Ma age separation between Lafayette and Yamato 000749 is robust.

**Supplementary Table 1:** Comparison of volume, volcanic lifespan, and eruption rates between Martian volcanoes and the Hawaiian islands, as representative of the largest terrestrial plume-derived volcanoes.

| Volcano | Relief (km) | Volcano lifespan (Ma) | Volume ($km^3$) | Average eruption rate ($km^3$ $Ma^{-1}$) |
|---|---|---|---|---|
| ***Mars:*** | | | | |
| Alba Patera | 5.8 [2] | 3320 (3500 to 180) [3] | 1.8E+06 [2] | 542 |
| Albor Tholus | 4.2 [2] | 2900 (3400 to 500) [3] | 2.9E+04 [2] | 10 |
| Arsia Mons | 11.7 [2] | 3410 (3540 to 130) [3] | 9.2E+05 [2] | 270 |
| Ascraeus Mons | 14.9 [2] | 3500 (3600 to 100) [3] | 1.1E+06 [2] | 314 |
| Elysium Mons | 12.6 [2] | 1100 (3700 to 2600) [3] | 2.0E+05 [2] | 182 |
| Hecates Tholus | 6.6 [2] | 3150 (3500 to 350) [3] | 6.7E+04 [2] | 21 |
| Olympus Mons | 21.9 [2] | 3650 (3800 to 150) [3] | 2.4E+06 [2] | 657 |
| Pavonis Mons | 8.4 [2] | 3480 (3560 to 80) [3] | 3.9E+05 [2] | 112 |
| Ulysses Patera | 1.5* [2] | 170 (3900 to 3730) [3] | 2.9E+03 [2] | 17 |
| Uranius Patera | 3.0* [2] | 200 (3700 to 3500) [3] | 3.5E+04 [2] | 175 |
| Uranius Tholus | 2.9* [2] | 540 (4040 to 3500) [3] | 3.4E+03 [2] | 6 |
| | | | | |
| ***Earth:*** | | | | |
| Kilauea | | 1† [4] | 3.16E+04 [5] | 31600 |
| Mauna Loa | | 1† [4] | 7.40E+04 [5] | 74000 |
| Mauna Kea | | 1† [4] | 4.19E+04 [5] | 41900 |
| Hualalai | | 1† [4] | 1.42E+04 [5] | 14200 |
| Kohala | | 1† [4] | 3.64E+04 [5] | 36400 |
| Mahukona | | 1† [4] | 1.35E+04 [5] | 13500 |
| Haleakala | | 1† [4] | 6.98E+04 [5] | 69800 |
| West Maui | | 1† [4] | 9.00E+03 [5] | 9000 |
| Kahoolawe | | 1† [4] | 2.63E+04 [5] | 26300 |
| Lanai | | 1† [4] | 2.11E+04 [5] | 21100 |
| East Molokai | | 1† [4] | 2.39E+04 [5] | 23900 |
| West Molokai | | 1† [4] | 3.03E+04 [5] | 30300 |
| Pauwela Ridge | | 1† [4] | 2.50E+03 [5] | 2500 |
| Koolau | | 1† [4] | 3.41E+04 [5] | 34100 |
| Waianae | | 1† [4] | 5.35E+04 [5] | 53500 |
| Kauai | | 1† [4] | 5.76E+04 [5] | 57600 |
| Niihau | | 1† [4] | 2.17E+04 [5] | 21700 |
| Kaula | | 1† [4] | 9.60E+03 [5] | 9600 |

* Volcanic edifices are partly buried, so the full relief is undetermined.
† Duration of shield-building stage only.

**Supplementary Table 2:** Compilation of chronologic results from the nakhlite meteorites.

| Meteorite & method | Age (Ma) | ± (Ma) * | MSWD † | Material | Ref. |
|---|---|---|---|---|---|
| **Lafayette** | | | | | |
| K-Ar | 0-670 | - | - | "iddingsite" | 6 |
| K-Ar | 1100 | 300 (?σ) | - | WR | 7 |
| U-Th/He | 830 | - | - | WR | 7 |
| $^{40}$Ar/$^{39}$Ar | 1330 | 30 | 1.6 | WR | 8 |
| $^{40}$Ar/$^{39}$Ar | 1300-1600 | - | - | WR | 9 |
| $^{40}$Ar/$^{39}$Ar | 1322 | 20 | 1.7 | WR | 10 |
| $^{40}$Ar/$^{39}$Ar | 1350 | 64 | 91 | Px | 11 |
| $^{40}$Ar/$^{39}$Ar | 1313 | 266 | 2052 | Ol | 11 |
| $^{40}$Ar/$^{39}$Ar | 1093 | 44 | 82 | Meso | 11 |
| $^{40}$Ar/$^{39}$Ar | 1306 | 14 | 27 | WR | 11 |
| Rb-Sr | 1260 | 70 (?σ) | - | WR, ol, px, Iddingsite | 12 |
| Sm-Nd | 1350 | 30 (?σ) | - | WR, ol, px, Iddingsite | 12 |
| U-Th-Pb | 1150 | 340 | | Apatite | 13 |
| **Governador Valadares** | | | | | |
| $^{40}$Ar/$^{39}$Ar | 1320 | 40 | 0.42 | WR | 14 |
| Rb-Sr | 1330 | 10 (?σ) | - | WR, px | 15 |
| Rb-Sr | >1200 | 50 (?2σ) | - | WR, ol, px, meso | 16 |
| Sm-Nd | 1370 | 20 (?2σ) | - | WR, ol, px, meso | 16 |
| **MIL 03346** | | | | | |
| K-Ar | 1750 | 260 (?σ) | - | WR | 17 |
| U-Th-He | 1020 | 150 (?σ) | - | WR | 17 |
| $^{40}$Ar/$^{39}$Ar | 1370 | - | - | WR | 18 |
| $^{40}$Ar/$^{39}$Ar | 1420 | 10 (?σ) | - | WR | 18 |
| $^{40}$Ar/$^{39}$Ar | 1373 | 105 | 37 | WR | 19 |
| $^{40}$Ar/$^{39}$Ar | 1368 | 83 | - | Mesostasis | 19 |
| $^{40}$Ar/$^{39}$Ar | 1334 | 54 | 0.26 | Px | 19 |
| $^{40}$Ar/$^{39}$Ar | 1343 | 34 | - | WR | 20 |
| $^{40}$Ar/$^{39}$Ar TG | 1367 | 30 | 22 | WR | 21 |
| Sm-Nd | 1360 | 30 (?σ) | - | WR, px, ol, meso | 22 |
| Rb-Sr | 1290 | 120 (?σ) | - | WR, px, ol, meso | 22 |
| Pb-Pb | ~1330 | - | - | WR, px | 23 |
| **MIL 090030** | | | | | |
| $^{40}$Ar/$^{39}$Ar | 738 | 33 (?σ) | - | Meso (plateau) | 24 |
| $^{40}$Ar/$^{39}$Ar | 769 | 11 (?σ) | - | Meso (isochron) | 24 |
| $^{40}$Ar/$^{39}$Ar | NA | NA | - | Meso (plateau) | 24 |
| $^{40}$Ar/$^{39}$Ar | 2350 | 590 (?σ) | - | Meso (isochron) | 24 |
| $^{40}$Ar/$^{39}$Ar | 1353 | 18 (?σ) | - | Meso (plateau) | 24 |
| $^{40}$Ar/$^{39}$Ar | 1421 | 37 (?σ) | - | Meso (isochron) | 24 |
| $^{40}$Ar/$^{39}$Ar | 1365 | 19 (?σ) | - | Meso (plateau) | 24 |
| $^{40}$Ar/$^{39}$Ar | 1438 | 13 (?σ) | - | Meso (isochron) | 24 |
| $^{40}$Ar/$^{39}$Ar | 1311 | 15 (?σ) | - | Meso (plateau) | 24 |
| $^{40}$Ar/$^{39}$Ar | 1300 | 28 (?σ) | - | Meso (isochron) | 24 |
| $^{40}$Ar/$^{39}$Ar | 1365 | 23 (?σ) | - | Meso (plateau) | 24 |
| $^{40}$Ar/$^{39}$Ar | 1388 | 8 (?σ) | - | Meso (isochron) | 24 |
| $^{40}$Ar/$^{39}$Ar | 1319 | 13 (?σ) | - | Meso (plateau) | 24 |
| $^{40}$Ar/$^{39}$Ar | 1423 | 18 (?σ) | - | Meso (isochron) | 24 |
| **MIL 090136** | | | | | |
| $^{40}$Ar/$^{39}$Ar | NA | NA | - | Meso (plateau) | 24 |
| $^{40}$Ar/$^{39}$Ar | 1544 | 14 (?σ) | - | Meso (isochron) | 24 |
| $^{40}$Ar/$^{39}$Ar | 1180 | 250 (?σ) | - | Meso (plateau) | 24 |
| $^{40}$Ar/$^{39}$Ar | 1310 | 550 (?σ) | - | Meso (isochron) | 24 |
| $^{40}$Ar/$^{39}$Ar | 1357 | 16 (?σ) | - | Meso (plateau) | 24 |
| $^{40}$Ar/$^{39}$Ar | 664 | 10 (?σ) | - | Meso (isochron) | 24 |
| $^{40}$Ar/$^{39}$Ar | 1335 | 4 (?σ) | - | Meso (plateau) | 24 |
| $^{40}$Ar/$^{39}$Ar | 1493 | 5 (?σ) | - | Meso (isochron) | 24 |
| $^{40}$Ar/$^{39}$Ar | 1327 | 26 (?σ) | - | Meso (plateau) | 24 |
| $^{40}$Ar/$^{39}$Ar | 1486 | 73 (?σ) | - | Meso (isochron) | 24 |
| $^{40}$Ar/$^{39}$Ar | 1351 | 11 (?σ) | - | Meso (plateau) | 24 |
| $^{40}$Ar/$^{39}$Ar | 1568 | 22 (?σ) | - | Meso (isochron) | 24 |
| $^{40}$Ar/$^{39}$Ar | 1352 | 19 (?σ) | - | Meso (plateau) | 24 |
| $^{40}$Ar/$^{39}$Ar | 1525 | 85 (?σ) | - | Meso (isochron) | 24 |
| **MIL 090132** | | | | | |
| $^{40}$Ar/$^{39}$Ar | 1418 | 7 (?σ) | - | Meso (plateau) | 24 |
| $^{40}$Ar/$^{39}$Ar | 1404 | 33 (?σ) | - | WR (isochron) | 24 |
| **Nakhla** | | | | | |
| K-Ar | 1500 | 300 (?σ) | - | WR? | 25 |
| K-Ar | 1400 | 300 (?σ) | - | WR | 7 |
| K-Ar | 1500 | 300 (?σ) | - | WR | 7 |
| U-Th-He | ~770 | - | - | WR | 7 |
| $^{40}$Ar/$^{39}$Ar | >1300 | - | - | WR | 8 |
| $^{40}$Ar/$^{39}$Ar | 1332 | 20 | 10 | WR | 10 |
| $^{40}$Ar/$^{39}$Ar | 1323 | 22 | 3.3 | WR | 10 |
| $^{40}$Ar/$^{39}$Ar | 1397 | 16 (?σ) | - | WR, acid treat WR | 26 |
| $^{40}$Ar/$^{39}$Ar | 1359 | 12 | - | WR (plateau) | 27 |
| $^{40}$Ar/$^{39}$Ar | 1357 | 14 | - | WR (isochron) | 27 |
| $^{40}$Ar/$^{39}$Ar | 1357 | 11 | 0.74 | WR | 19 |
| $^{40}$Ar/$^{39}$Ar | 1328 | 52 | - | WR, ol, px | 20 |
| $^{40}$Ar/$^{39}$Ar | 1415 | 134 | - | Meso | 11 |
| $^{40}$Ar/$^{39}$Ar | 1399 | 56 | - | Px (a) | 11 |
| $^{40}$Ar/$^{39}$Ar | 1389 | 70 | 499 | Px (b) | 11 |
| $^{40}$Ar/$^{39}$Ar | 1418 | 36 | 53 | WR | 11 |
| $^{40}$Ar/$^{39}$Ar TG | 1345 | 20 (?2σ) | - | WR, meso, ol, px | 28 |
| $^{40}$Ar/$^{39}$Ar TG | 2030 | 50 | 15 | WR | 21 |
| $^{40}$Ar/$^{39}$Ar TG | 1378 | 30 | 716 | WR | 21 |
| $^{40}$Ar/$^{39}$Ar TG | 1824 | 270 | - | Ol | 21 |
| $^{40}$Ar/$^{39}$Ar TG | 1342 | 54 | 19 | Ol | 21 |
| $^{40}$Ar/$^{39}$Ar TG | 2102 | 68 | 26 | Px | 21 |
| $^{40}$Ar/$^{39}$Ar TG | 1364 | 30 | 191 | Px | 21 |
| $^{40}$Ar/$^{39}$Ar TG | 1374 | 30 | 59 | Mesostasis | 21 |
| $^{40}$Ar/$^{39}$Ar TG | 1365 | 28 | 108 | Px | 21 |
| $^{40}$Ar/$^{39}$Ar TG | 1393 | 34 | 26 | ol | 21 |
| $^{40}$Ar/$^{39}$Ar TG | 1373 | 32 | 6.2 | Bulk 1 | 21 |
| $^{40}$Ar/$^{39}$Ar TG | 1405 | 28 | 53 | Bulk 2 | 21 |
| $^{40}$Ar/$^{39}$Ar TG | 1348 | 28 | 23 | WR (water etched) | 21 |
| $^{40}$Ar/$^{39}$Ar TG | 1338 | 34 | 64 | WR (acid etched) | 21 |
| Rb-Sr | 4370 | - | - | WR (model age) | 29 |
| Rb-Sr | 1242 | 10 | - | WR, px, plag, ol | 30 |
| Rb-Sr | 1370 | 20 (?σ) | - | WR, px, plag, ol | 31 |
| Sm-Nd | 1260 | 70 | - | WR, px, pl | 32 |
| Sm-Nd | 1380 | 70 (?σ) | - | WR, px, ol, meso | 33 |
| U-Pb | 1280 | 50 | - | WR, px, ol | 32 |
| U-Th-Pb | 1240 | 110 | - | WR, px, ol | 32 |
| Pb-Pb | ~1300 | - | - | WR, leached WR | 34 |
| Re-Os | 1405 | 19 (?σ) | - | WR | 35 |
| **NWA 998** | | | | | |
| K-Ar | 1350 | - | - | WR | 36 |
| $^{40}$Ar/$^{39}$Ar | 1332 | 16 | - | WR | 27 |
| $^{40}$Ar/$^{39}$Ar | 1411 | 4 (?2σ) | - | Ol, px, feld | 28 |
| $^{40}$Ar/$^{39}$Ar | 1334 | 11 | 0.62 | Plag | 19 |
| $^{40}$Ar/$^{39}$Ar TG | 1450 | 54 | 1.06 | plag | 21 |
| $^{40}$Ar/$^{39}$Ar TG | 1307 | 64 | 5.10 | Px | 21 |
| $^{40}$Ar/$^{39}$Ar TG | 1138 | 74 | 1.90 | Px | 21 |
| $^{40}$Ar/$^{39}$Ar TG | 1347 | 74 | 2.30 | Px | 21 |
| $^{40}$Ar/$^{39}$Ar TG | 1364 | 174 | 1.60 | Px | 21 |
| $^{40}$Ar/$^{39}$Ar TG | 3039 | 142 | 13 | Olivine | 21 |
| **NWA 5790** | | | | | |
| Sm-Nd | 1380 | 100 (?σ) | - | WR, px, meso | 33 |
| **NWA 10153** | | | | | |
| Sm-Nd | 1419 | 56 (?σ) | 0.57 | WR, px, plag | 37 |
| Lu-Hf | 1360 | 33 (?σ) | 0.53 | WR, px, plag | 37 |
| **Yamato 000593/749** | | | | | |
| $^{40}$Ar/$^{39}$Ar | <1359 | 20 | - | WR isochron | 27,38 |
| $^{40}$Ar/$^{39}$Ar | 1405 | 107 | 86 | WR | 19 |
| $^{40}$Ar/$^{39}$Ar | 1397 | 91 | 163 | Plag | 19 |
| $^{40}$Ar/$^{39}$Ar | 1367 | 7 | 0.44 | Plag (<16 % $^{39}$Ar) | 19 |
| $^{40}$Ar/$^{39}$Ar | 1416 | 116 | 5 | Px | 19 |
| Pb-Pb | ~1330 | - | - | Px, WR | 23 |
| Rb-Sr | 1300 | 20 | - | WR, px, ol, meso | 38,39 |
| Sm-Nd | 1310 | 30 (2?σ) | - | WR, px, meso | 38,39 |
| U-Th-Pb | 1530 | 460 | - | Apatite SHRIMP | 13 |

Ages listed are those originally reported. Abbreviations: TG = total-gas, WR = whole-rock, px = pyroxene, ol = olivine, plag = plagioclase, feld = feldspar, meso = mesostasis.

* 2σ analytical uncertainty. "?" indicates where data not specified as 1σ or 2σ.

† Values listed in original reference, or calculated using IsoPlot 3.0 if sufficiently detailed data was reported.

**Supplementary Table 3:** Cosmogenic exposure age measurements on unirradiated fragments.

| Meteorite | Mass (mg) | Ca | ±1σ | Fe | ±1σ | Ni | ±1σ | Ti | ±1σ | Cr | ±1σ | Mn | ±1σ | K | ±1σ | $^{38}$Ar Prod. rate | ±1σ | $^{38}$Ar/$^{36}$Ar | ± (1σ) | $^{38}$Ar(cos) (moles) | ± (1σ) | Exposure Age (Ma) | ± 2σ (full external) |
|---|---|---|---|---|---|---|---|---|---|---|---|---|---|---|---|---|---|---|---|---|---|---|---|
| Lafayette | 18.30 | 9.60 | 0.50 | 16.80 | 1.70 | 0.0096 | 0.0015 | 0.254 | 0.080 | 0.128 | 0.007 | 0.388 | 0.003 | 0.0900 | 0.0130 | 19.58 | 0.51 | 1.582 | 0.782 | 1.651E-14 | 1.4E-15 | **10.3** | **1.0** |
| MIL 03346 | 5.044 | 8.89 | 0.07 | 15.48 | 0.12 | 0.0038 | 0.0001 | 0.476 | 0.004 | 0.093 | 0.001 | 0.366 | 0.003 | 0.1874 | 0.0007 | 18.50 | 0.10 | 1.483 | 0.042 | 4.167E-15 | 3.1E-17 | **12.3** | **1.2** |
| Nakhla (a) | 4.333 | 8.16 | 0.08 | 22.08 | 0.17 | 0.0081 | 0.0002 | 0.170 | 0.003 | 0.151 | 0.001 | 0.500 | 0.007 | 0.0567 | 0.0008 | 17.42 | 0.12 | 1.506 | 0.028 | 3.784E-15 | 2.7E-17 | **11.2** | **1.1** |
| Nakhla (b) | 12.68 | 10.50 | 0.50 | 16.00 | 1.20 | 0.0090 | - - - - - | 0.202 | 0.025 | 0.177 | 0.023 | 0.382 | 0.031 | 0.1070 | 0.0190 | 20.12 | 0.48 | 1.086 | 0.027 | 1.225E-14 | 2.2E-16 | **10.2** | **1.0** |
| NWA 5790 | 4.124 | 6.87 | 0.07 | 20.33 | 0.11 | 0.0055 | 0.0001 | 0.333 | 0.002 | 0.049 | 0.001 | 0.456 | 0.004 | 0.3037 | 0.0042 | 15.63 | 0.10 | 1.537 | 0.056 | 3.365E-15 | 2.4E-17 | **9.6** | **1.0** |
| Yamato 000593 | 4.952 | 6.41 | 0.04 | 25.14 | 0.14 | 0.0052 | 0.0001 | 0.134 | 0.002 | 0.108 | 0.001 | 0.559 | 0.004 | 0.0243 | 0.0004 | 14.44 | 0.07 | 1.487 | 0.033 | 3.586E-15 | 2.8E-17 | **11.3** | **1.1** |
| Yamato 000749 | 4.395 | 11.14 | 0.06 | 12.01 | 0.04 | 0.0050 | 0.0001 | 0.162 | 0.001 | 0.194 | 0.001 | 0.332 | 0.001 | 0.0777 | 0.0013 | 21.82 | 0.08 | 1.544 | 0.061 | 4.366E-15 | 3.4E-17 | **10.2** | **1.0** |

When possible, the chemical compositions of the fragments analysed for cosmogenic noble gases were measured by ICP-MS at Lawrence Livermore National Laboratory (LLNL) to avoid biasing results due to chemical heterogeneities that may be retained by small sample sizes. Aliquots of larger fragments analysed in Pt-Ir packets at LLNL (Nakhla (b) and Lafayette; see methods) were not quantitatively recovered from the packets following laser heating. As such, published chemical data[40] were used to calculate production rates for these aliquots.

$^{38}$Ar production rates given in $10^{-10}$ $cm^3$, STP $g^{-1}$ $Ma^{-1}$, calculated following [41]. Due to the ~10 % experimental uncertainty (2σ) in $^{38}$Ar production rates[41], the 2σ full-external uncertainty for all cosmogenic exposure ages is ~ ± 10%. Mass spectrometer sensitivity: SUERC: ~6.5 x $10^{-15}$ mol $Volt^{-1}$; LLNL: ~3.1 x $10^{-20}$ mol Counts Per $Second^{-1}$.

($^{38}$Ar/$^{36}$Ar)cos = 1.54, ref. [42]

($^{38}$Ar/$^{36}$Ar)trap = 0.244, ref. [43]

**Supplementary Table 4:** Compilation of proposed source craters for Martian meteorites, and calculated eruption rates based on meteorite excavation depth.

| Crater name or latitude + longitude | Comment | Crater diameter (km) | Bolide radius (m) | Excavated area (m) | Excavation depth (m) | Eruption rate (m $Ma^{-1}$) |
|---|---|---|---|---|---|---|
| **Craters suggested for nakhlites:** | | | | | | |
| 76.5°E, 15.5°N [44] | Not on Amazonian volcanic terrain [45] | 14x22 | 650 | 1910 | 130 | 1.4 |
| 126.057°E, 20.154°N [46] | Not on Amazonian volcanic terrain [45] | 12.6 | 430 | 1280 | 85 | 0.9 |
| 122.242°E, 23.971°N [46] | Not on Amazonian volcanic terrain [45] | 10.9 | 360 | 1080 | 72 | 0.8 |
| **130.799°E, 29.674°N [46]** | **Amazonian volcanic terrain[45] & >6 km cutoff [47]** | **6.5** | **200** | **600** | **40** | **0.4** |
| 121.555°E, 29.004°N [46] | Below 6 km diameter cutoff [47] | 4.5 | 130 | 400 | 26 | 0.3 |
| 128.717°E, 26.346°N [46] | Below 6 km diameter cutoff [47] | 3.7 | 110 | 320 | 21 | 0.2 |
| 226.621°E, 54.469°N [46] | Below 6 km diameter cutoff [47] | 2.3 | 60 | 190 | 12 | 0.1 |
| **Other craters:** | | | | | | |
| Mojave [48] | On early Noachian terrain [48] | 55 | 2270 | 6800 | 453 | 4.9 |
| Zunil [49,50] | Suggested as source of shergottites [49] | 10.1 | 330 | 990 | 66 | 0.7 |
| Tomini [49] | Not on Amazonian volcanic terrain [45] | 7.4 | 230 | 700 | 47 | 0.5 |
| Gratteri [49] | Not on Amazonian volcanic terrain [45] | 6.9 | 220 | 645 | 43 | 0.5 |
| 155.5°E, 18.1°N [49] | Below 6 km diameter cutoff [47] | 5.7 | 170 | 520 | 35 | 0.4 |
| Tomini B [49] | Below 6 km diameter cutoff [47] | 4.2 | 120 | 370 | 25 | 0.3 |
| Zumba [49] | Below 6 km diameter cutoff [47] | 3.3 | 93 | 280 | 19 | 0.2 |
| Dilly [49] | Below 6 km diameter cutoff [47] | 2.0 | 53 | 160 | 10 | 0.1 |
| 159.2°W, 15.5°N [49] | Below 6 km diameter cutoff [47] | 1.5 | 38 | 115 | 8 | 0.1 |

These localities are all youthful rayed craters on Mars. We have compiled craters suggested as the source for the shergottites as well as the nakhlites, because the uncertainties determining the age of the Martian surface via crater counting methods may mean that some of these craters previously suggested for the shergottites (terrain < 700 Ma, late Amazonian) could have impacted mid-Amazonian rocks of nakhlite age (1300–1400 Ma). Craters < 6 km wide have also been proposed for the nakhlites[46,49], but here we use the cutoff diameter of 6 km proposed by ref. [47], as a terrain of ca. 1.3 Ga age is expected to have a significant regolith overburden, which impedes meteorite launching into space, and requires a larger minimum crater diameter than for younger terrains with less regolith. The ~10 km maximum reflects the lack of larger craters on appropriate Martian volcanic terrain; indeed, large and young (<~10 Ma) craters are scarce in the inner solar system. We have not compiled craters suggested by earlier workers that used Viking satellite imagery e.g., 51,52.

The calculated depths and areas are more than sufficient to sample the set of at least four igneous units indicated by the $^{40}Ar/^{39}Ar$ results. Sampling of multiple flows is especially plausible if the nakhlites were emplaced as a remobilized cumulate crystal mush which lost interstitial melt via lava breakouts[53], as this emplacement model allows for thinner lava flows than the cumulate model of nakhlite crystallization[54,55].

The bolide radius was calculated using equations 21 and 27 from ref. 56 for a bolide density of 3000 kg $m^{-3}$ (i.e., equivalent to an ordinary chondritic impactor, the most common type of meteorite), an impact angle of 45 degrees (the most probable angle[57]), impactor velocity of 10 km $s^{-1}$, into basaltic crystalline rock with density of 2800 kg $m^{-3}$, and Martian surface gravity of 3.711 m $s^{-2}$. The excavated area and depth were calculated using a maximum spallation depth for excavation of Martian meteorites of 0.2 times the impacting bolide radius and maximum area of 3 times the bolide radius[58].

**Supplementary Table 5:** Values used in the $^{40}Ar/^{39}Ar$ data regression procedures.

| | Value | Reference |
|---|---|---|
| $(^{40}Ar/^{36}Ar)_{Earth\ atmosphere}$ | 298.56 ± 0.31 | For mass discrimination calculations only[59] |
| $^{40}K/K$ | 1.167 E-4 mol mol$^{-1}$ | [60] |
| $^{40}K$ λε | (5.757 ± 0.016) E-11 a$^{-1}$ | [61] |
| $^{40}K$ $\lambda_\beta$ | (4.9548 ± 0.0134) E-10 a$^{-1}$ | [61] |
| $^{40}K$ λ total | (5.5305 ± 0.0135) E-10 a$^{-1}$ | [61] |
| $^{37}Ar$ λ | 0.01983 ± .0000226 days$^{-1}$ | [62] |
| $^{39}Ar$ λ | (7.055 ± 0.039) E-6 days$^{-1}$ | [63] |
| $^{36}Cl$ $\lambda_\beta$ | (6.1817 ± 0.040) E-9 days$^{-1}$ | [64] |
| | | |
| Reactor production ratios and interfering isotope production ratios: | | |
| $(^{36}Cl/^{38}Cl)_{Cl}$ | 263 ± 2 | [64] |
| $(^{38}Ar/^{37}Ar)_{Ca}$ | (1.96 ± 0.08) E-05 | [65] |
| $(^{38}Ar/^{39}Ar)_{K}$ | (1.22 ± 0.01) E-02 | [65] |
| $(^{40}Ar/^{39}Ar)_{K}$ | (7.30 ± 0.92) E-04 | [65] |
| $(^{37}Ar/^{39}Ar)_{K}$ | (2.24 ± 0.16) E-04 | [65] |
| $(^{39}Ar/^{37}Ar)_{Ca}$ | (6.95 ± 0.09) E-04 | [65] |
| $(^{36}Ar/^{37}Ar)_{Ca}$ | (2.65 ± 0.02) E-04 | [65] |

**Supplementary References:**


1 Cassata, W. S. & Borg, L. E. A new approach to cosmogenic corrections in $^{40}Ar/^{39}Ar$ chronometry: implications for the ages of Martian meteorites. *Geochim. Cosmochim. Acta* **187**, 279-293 (2016).

2 Plescia, J. B. Morphometric properties of Martian volcanoes. *J. Geophys. Res.* **109**, E03003, doi:10.1029/2002JE002031 (2004).

3 Werner, S. C. The global Martian volcanic evolutionary history. *Icarus* **201**, 44-68 (2009).

4 Sharp, W. D. & Renne, P. R. The $^{40}Ar/^{39}Ar$ dating of core recovered by the Hawaii Scientific Drilling Project (phase 2), Hilo, Hawaii. *Geochem., Geophys., Geosyst.* **6**, Q04G17, doi:10.1029/2004GC000846 (2005).

5 Robinson, J. E. & Eakins, B. W. Calculated volumes of individual shield volcanoes at the young end of the Hawaiian Ridge. *J. Volcanol. Geotherm. Res.* **151**, 309-317 (2006).

6 Swindle, T. D. *et al.* Noble gases in iddingsite from the Lafayette meteorite: Evidence for liquid water on Mars in the last few hundred million years. *Meteoritics & Planet. Sci.* **35**, 107-115 (2000).

7 Ganapathy, R. & Anders, E. Ages of calcium-rich achondrites-II. Howardites, nakhlites, and the Angra dos Reis angrite. *Geochim. Cosmochim. Acta* **33**, 775-787 (1969).

8 Podosek, F. A. Thermal history of the nakhlites by the $^{40}Ar$-$^{39}Ar$ method. *Earth Planet. Sci. Lett.* **19**, 135-144 (1973).

9 Podosek, F. A. & Huneke, J. C. Argon 40-argon 39 chronology of four calcium-rich achondrites. *Geochim. Cosmochim. Acta* **37**, 667-684 (1973).

10 Swindle, T. D. & Olson, E. K. $^{40}Ar$-$^{39}Ar$ studies of whole rock nakhlites: evidence for the timing of formation and aqueous alteration on Mars. *Meteoritics & Planet. Sci.* **39**, 755-766 (2004).

11 Korochantseva, E. V. *et al.* $^{40}Ar$-$^{39}Ar$ and cosmic-ray exposure ages of nakhlites-Nakhla, Lafayette, Governador Valadares-and Chassigny. *Meteoritics & Planet. Sci.* **46**, 1397-1417 (2011).

12 Shih, C. Y., Nyquist, L. E., Reese, Y. & Wiesmann, H. The Chronology of the Nakhlite, Lafayette: Rb-Sr and Sm-Nd isotopic ages. *Lunar Planet. Sci. Conf.* **XXIX,** 1145 (1998).

13 Terada K. & Y., S. Ion microprobe U-Th-Pb dating and REE analyses of phosphates in the nakhlites Lafayette and Yamato-000593/000749. *Meteoritics & Planet. Sci.* **39**, 2033-2041 (2004).

14 Bogard, D. D. & Husain, L. A new 1.3 aeon-young achondrite. *Geophys. Res. Lett.* **4**, 69-71 (1977).

15 Wooden, J. L. *et al.* Radiometric ages for the achondrites Chervony Kut, Governador Valadares, and Allan Hills 77005. *Lunar Planet. Sci. Conf.* **X,** 1379–1381 (1979).

16 Shih, C. Y., Nyquist, L. E. & Wiesmann, H. Samarium-neodymium and rubidium-strontium systematics of nakhlite Governador Valadares. *Meteoritics & Planet. Sci.* **34**, 647-655 (1999).

17 Murty, S. V. S., Mahajan, R. R., Goswami, J. N. & Sinha, N. Noble gases and nuclear tracks in the nakhlite MIL 03346. *Lunar Planet. Sci. Conf.* **XXXVI,** 1280 (2005).
18 Bogard, D. D. & Garrison, D. H. Ar-Ar dating of Martian chassignites, NWA2737 and Chassigny, and nakhlite MIL03346. *Lunar Planet. Sci. Conf.* **XXXVII,** 1108 (2006).
19 Park, J., Garrison, D. H. & Bogard, D. D. $^{39}$Ar-$^{40}$Ar ages of martian nakhlites. *Geochim. Cosmochim. Acta* **73**, 2177-2189 (2009).
20 Cassata, W. S., Shuster, D. L., Renne, P. R. & Weiss, B. P. Evidence for shock heating and constraints on Martian surface temperatures revealed by $^{40}$Ar/$^{39}$Ar thermochronometry of Martian meteorites. *Geochim. Cosmochim. Acta* **74**, 6900-6920 (2010).
21 Cartwright, J. A., Gilmour, J. D. & Burgess, R. Martian fluid and Martian weathering signatures identified in Nakhla, NWA 998 and MIL 03346 by halogen and noble gas analysis. *Geochim. Cosmochim. Acta* **105**, 255-293 (2013).
22 Shih, C. Y., Nyquist, L. E. & Reese, Y. Rb-Sr and Sm-Nd isotopic studies of Antarctic nakhlite MIL 03346. *Lunar Planet. Sci. Conf.* **XXXVII,** 1701 (2006).
23 Bouvier, A., Blichert-Toft, J. & Albarède, F. Martian meteorite chronology and the evolution of the interior of Mars. *Earth Planet. Astrophys.* **280**, 285-295 (2009).
24 Park, J. *et al.* $^{40}$Ar/$^{39}$Ar ages of nakhlites Miller Range (MIL) 090030, 090032 and 090136. *Lunar Planet. Sci. Conf.* **XLVII,** 1821 (2016).
25 Stauffer, H. On the production rates of rare gas isotopes in stone meteorites. *J. Geophys. Res.* **67**, 2023-2028 (1962).
26 Burgess, R., Holland, G., Fernandes, V. & Turner, G. New Ar-Ar data on Nakhla minerals. *Goldschmidt Conf.* 266 (2000).
27 Garrison, D. H. & Bogard, D. D. Ar-Ar Ages of Nakhlites Y000593, NWA998, and Nakhla and CRE age of NWA998. *Lunar Planet. Sci. Conf.* **XXXVI,** 1137 (2005).
28 Cartwright, J. A., Burgess, R., Gilmour, J. D. & Turner, G. Ar and Cl-bearing hydrous fluids in Nakhlites. *Lunar Planet. Sci. Conf.* **XLI** #2214 (2010).
29 Pinson, W. H., Jr,, Schnetzler, C. C., Beiser, E., Fairbairn, H. W. & Hurley, P. M. Rb-Sr age of stony meteorites. *Geochim. Cosmochim. Acta* **29**, 455-466 (1965).
30 Gale, N. H., Arden, J. W. & Hutchison, R. The chronology of the Nakhla achondritic meteorite. *Earth Planet. Sci. Lett.* **26**, 195-206 (1975).
31 Papanastassiou, D. A. & Wasserberg, G. J. Evidence for late formation and young metamorphism in the achondrite Nakhla. *Geophys. Res. Lett.* **1**, 23-26 (1974).
32 Nakamura, N., Unruh, D. M., Tatsumoto, M. & Hutchison, R. Origin and evolution of the Nakhla meteorite inferred from the Sm-Nd and U-Pb systematics and REE, Ba, Sr, Rb and K abundances. *Geochim. Cosmochim. Acta* **46**, 1555-1573 (1982).
33 Shih C.-Y., Nyquist L.E., Reese Y. & Jambon A. Sm- Nd isotopic studies of two nakhlites, NWA 5790 and Nakhla. *Lunar Planet. Sci. Conf.* **XLI,** 1367 (2010).
34 Bouvier, A., Blichert-Toft, J., Vervoort, J. D. & Albarède, F. The age of SNC meteorites and the antiquity of the Martian surface. *Earth Planet. Sci. Lett.* **240**, 221-233 (2005).
35 Walker R. J. *et al.* $^{187}$Re-$^{187}$Os isotopic studies of SNC meteorites: An update *Lunar Planet. Sci. Conf.* **XXXIII,** 1042 (2002).
36 Marty, B. *et al.* Noble gases in new SNC meteorites NWA 817 and NWA 480. *Meteoritics & Planet. Sci.* **36**, A122-A123 (2001).
37 Righter, K., Lapen, T. J., Andreasen, R. & Irving, A. J. Sm-Nd and Lu-Hf systematics of NWA 10153. *Goldschmidt Conf.* **26,** 2632 (2016).
38 Misawa, K. *et al.* Rb-Sr, Sm-Nd and Ar-Ar isotopic systematics of Antarctic nakhlite Yamato 000593. *Antarctic Meteorite Research* **18**, 133-151 (2005).
39 Misawa, K., Shih, C.-Y., Wiesmann, H. & Nyquist, L. E. Crystallization and alteration ages of the Antarctic nakhlite Yamato 000593. *Lunar Planet. Sci. Conf.* **XXXIV,** 1556 (2003).
40 Lodders, K. A survey of shergottite, nakhlite and chassigny meteorites whole-rock compositions. *Meteoritics & Planet. Sci.* **33**, A183-A190 (1998).
41 Eugster, O. & Michel, T. Common asteroid break-up events of eucrites, diogenites, and howardites and cosmic-ray production rates for noble gases in achondrites. *Geochim. Cosmochim. Acta* **59**, 177-199 (1995).
42 Wieler, R. Cosmic-ray-produced noble gases in meteorites. *Rev. Mineralogy and Geochem.* **47**, 125-170 (2002).
43 Wiens, R. C., Becker, R. H. & Pepin, R. O. The case for a martian origin of the shergottites, II. Trapped and indigenous gas components in EETA 79001 glass. *Earth Planet. Sci. Lett.* **77**, 149-158 (1986).

44 Harvey, R. P. & Hamilton, V. E. Syrtis Major as the source region of the nakhlite/chassigny group of Martian meteorites: implications for the geological history of Mars. *Lunar Planet. Sci. Conf.* **XXXVI,** 1019 (2005).
45 Tanaka, K. L. *et al.* Geologic map of Mars. *U.S. Geological Survey Scientific Investigations* **Map 3292** (2014).
46 Kereszturi, A. & Chatzitheodoridis, E. Searching for the source crater of nakhlite meteorites. *Orig. Life Evol. Biosph.* **46** (2016).
47 Head, J. N., Melosh, H. J. & Ivanov, B. A. Martian meteorite launch: high-speed ejecta from small craters. *Science* **298**, 1752-1756 (2002).
48 Werner, S. C., Ody, A. & Poulet, F. The source crater of Martian shergottite meteorites. *Science* **343**, 1343-1346 (2014).
49 Tornabene, L. L. *et al.* Identification of large (2–10 km) rayed craters on Mars in THEMIS thermal infrared images: implications for possible Martian meteorite source regions. *J. Geophys. Res.* **111**, E10006, doi:10.1029/2005je002600 (2006).
50 McEwen, A. *et al.* The rayed crater Zunil and interpretations of small impact craters on Mars. *Icarus* **176**, 351-381 (2005).
51 Mouginis-Mark, P. J., McCoy, T. J., Taylor, G. J. & Keil, K. Martian parent craters for the SNC meteorites. *J. Geophys. Res.* **97**, 10213-10225 (1992).
52 Treiman, A. H. S ≠ NC: Multiple source areas for Martian meteorites. *J. Geophys. Res.* **100**, 5329-5340 (1995).
53 Corrigan, C. M., Velbel, M. A. & Vicenzi, E. P. Modal abundances of pyroxene, olivine, and mesostasis in nakhlites: Heterogeneity, variation, and implications for nakhlite emplacement. *Meteoritics & Planet. Sci.* **50**, 1497-1511 (2015).
54 Mikouchi, T., Makishima, J., Kurihara, T., Hoffmann, V. H. & Miyamoto, M. Relative burial depth of Nakhlites revisited. *Lunar Planet. Sci. Conf.* **XLIII,** 2363 (2012).
55 Mikouchi, T., Miyamoto, M., Koizumi, E., Makishima, J. & McKay, G. Relative burial depths of Nakhlites: an update. *Lunar Planet. Sci. Conf.* **XXXVII,** 1865 (2006).
56 Collins, C. S., Melosh, H. J. & Marcus, R. A. Earth Impact Effects Program: a web-based computer program for calculating the regional environmental consequences of a meteoroid impact on Earth. *Meteoritics & Planet. Sci.* **40**, 817-840 (2005).
57 Artemieva, N. & Ivanov, B. Launch of martian meteorites in oblique impacts. *Icarus* **171**, 84-101 (2004).
58 Fritz, J., Artemieva, N. & Greshake, A. Ejection of Martian meteorites. *Meteoritics & Planet. Sci.* **40**, 1393-1411 (2005).
59 Lee, J.-Y. *et al.* A redetermination of the isotopic abundances of atmospheric Ar. *Geochim. Cosmochim. Acta* **70**, 4507-4512 (2006).
60 Steiger, R. H. & Jäger, E. Subcommission on geochronology: convention on the use of decay constants in geo- and cosmochemistry. *Earth Planet. Sci. Lett.* **36**, 359-362 (1977).
61 Renne, P. R., Balco, G., Ludwig, K. R., Mundil, R. & Min, K. Response to the comment by W.H. Schwarz et al. on "Joint determination of $^{40}$K decay constants and $^{40}$Ar*/$^{40}$K for the Fish Canyon sanidine standard, and improved accuracy for $^{40}$Ar/$^{39}$Ar geochronology" by Paul R. Renne et al. (2010). *Geochim. Cosmochim. Acta* **75**, 5097-5100 (2011).
62 Renne, P. & Norman, E. Determination of the half-life of $^{37}$Ar by mass spectrometry. *Phys. Rev. C* doi:10.1103/PhysRevC.63.047302 **63** (2001).
63 Stoenner, R. W., Schaeffer, O. A. & Katcoff, S. Half-lives of Argon-37, Argon-39, and Argon-42. *Science* **148**, 1325-1328 (1965).
64 Renne, P. R., Sharp, Z. D. & Heizler, M. T. Cl-derived argon isotope production in the CLICIT facility of OSTR reactor and the effects of the Cl-correction in $^{40}$Ar/$^{39}$Ar geochronology. *Chem. Geol.* **255**, 463-466 (2008).
65 Renne, P. R., Knight, K. B., Nomade, S., Leung, K.-N. & Lou, T.-P. Application of deuteron-deuteron (D-D) fusion neutrons to $^{40}$Ar/$^{39}$Ar geochronology. *Applied Rad. Isotopes* **62**, 25-32 (2005).
